# Characterization of peer support services for substance use disorders in 11 US emergency departments in 2020: findings from a NIDA clinical trials network site selection process

**DOI:** 10.1186/s13722-024-00453-x

**Published:** 2024-04-08

**Authors:** Lindsey K Jennings, Laura Lander, Tricia Lawdahl, Erin A. McClure, Angela Moreland, Jenna L. McCauley, Louise Haynes, Timothy Matheson, Richard Jones, Thomas E. Robey, Sarah Kawasaki, Phillip Moschella, Amer Raheemullah, Suzette Miller, Gina Gregovich, Deborah Waltman, Kathleen T. Brady, Kelly S. Barth

**Affiliations:** 1 Department of Emergency Medicine, Medical University of South Carolina, 169 Ashley Avenue, MSC 300, Charleston, SC 29425 USA; 2 Department of Behavioral Medicine & Psychiatry, West Virginia University, Morgantown, WV USA; 3 Faces and Voices of Recovery (FAVOR) Upstate, Greenville, SC USA; 4 Department of Psychiatry and Behavioral Sciences, Medical University of South Carolina, Charleston, SC USA; 5 Center On Substance Use and Health, San Francisco Department of Public Health, San Francisco, CA USA; 6 Yourturn Health Inc, Coppell, TX USA; 7 Providence Regional Medical Center Everett, Washington State University, Everett, WA USA; 8 Departments of Psychiatry and Internal Medicine, Penn State Health, Hershey, PA USA; 9 Department of Emergency Medicine, University of South Carolina School of Medicine, Greenville, SC USA; 10 Department of Psychiatry and Behavioral Sciences, Stanford University School of Medicine, Stanford, CA USA; 11 Mercy Health - St. Elizabeth Youngstown Hospital, Youngstown, OH USA; 12 Department of Internal Medicine, University of Utah, Salt Lake City, UT USA; 13 Deaconess Hospital, MultiCare Health System, Spokane, WA USA

**Keywords:** Emergency department, Substance use disorders, Peer Support Specialists

## Abstract

**Introduction:**

Emergency departments (ED) are incorporating Peer Support Specialists (PSSs) to help with patient care for substance use disorders (SUDs). Despite rapid growth in this area, little is published regarding workflow, expectations of the peer role, and core components of the PSS intervention. This study describes these elements in a national sample of ED-based peer support intervention programs.

**Methods:**

A survey was conducted to assess PSS site characteristics as part of site selection process for a National Institute on Drug Abuse (NIDA) Clinical Trials Network (CTN) evaluating PSS effectiveness, Surveys were distributed to clinical sites affiliated with the 16 CTN nodes. Surveys were completed by a representative(s) of the site and collected data on the PSS role in the ED including details regarding funding and certification, services rendered, role in medications for opioid use disorder (MOUD) and naloxone distribution, and factors impacting implementation and maintenance of ED PSS programs. Quantitative data was summarized with descriptive statistics. Free-text fields were analyzed using qualitative content analysis.

**Results:**

A total of 11 surveys were completed, collected from 9 different states. ED PSS funding was from grants (55%), hospital funds (46%), peer recovery organizations (27%) or other (18%). Funding was anticipated to continue for a mean of 16 months (range 12 to 36 months). The majority of programs provided “general recovery support (81%) Screening, Brief Intervention, and Referral to Treatment (SBIRT) services (55%), and assisted with naloxone distribution to ED patients (64%). A minority assisted with ED-initiated buprenorphine (EDIB) programs (27%). Most (91%) provided services to patients after they were discharged from the ED. Barriers to implementation included lack of outpatient referral sources, barriers to initiating MOUD, stigma at the clinician and system level, and lack of ongoing PSS availability due to short-term grant funding.

**Conclusions:**

The majority of ED-based PSSs were funded through time-limited grants, and short-term grant funding was identified as a barrier for ED PSS programs. There was consistency among sites in the involvement of PSSs in facilitation of transitions of SUD care, coordination of follow-up after ED discharge, and PSS involvement in naloxone distribution.

**Supplementary Information:**

The online version contains supplementary material available at 10.1186/s13722-024-00453-x.

## Introduction

Overdose death rates in the United States increased from 6.1 per 100,000 in 1999 to 28.3 per 100,000 in 2021 [14]. Concurrent with this surge in overdose deaths, the peer support specialist (PSS) workforce experienced dramatic growth. There is growing evidence supporting the effectiveness of peer-based recovery interventions, including on outcomes including reduced substance use rates, higher levels of treatment retention, increased medications for opioid use disorder (MOUD) initiation, increased involvement in 12-step programs, increased satisfaction with treatment, increased naloxone distribution, and reduced emergency department (ED) visits and hospitalizations [1, 10, 12, 17, 18, 23, 26, 32]. In 2015, SAMHSA endorsed PSS as an “evidence-based practice” and developed 12 core competencies for PSSs in behavioral health settings [28]. In 2023, SAMHSA released National Model Standards for Peer Support Certification [29].

In 2016, funding from the 21st Century Cures Act further supported the growth and implementation of the PSS workforce. This funding, often awarded in the form of block or discretionary grants, generated $1 billion dollars to support projects to address the opioid overdose crisis, disseminated primarily via State Targeted Response (STR) grants (HHS.gov/opioids) and State Opioid Response (SOR) grants. One of the requests of these grants was to increase PSSs in ED settings [34; [22]; SAMHSA.gov/grants), and several ED-based PSS models implemented during this time demonstrated positive outcomes related to patient engagement and successful linkages to treatment [3, 7, 22, 25, 32, 33], and [4].

As part of the site selection process for a NIDA Clinical Trials Network (CTN) multi-site national study, 11 ED sites from 9 states provided survey responses characterizing PSS in their ED in 2020. We provide a description of implemented peer support services reported in the Site Selection Surveys (SSS) as they existed in those EDs in 2020 with the aim of comparing themes, similarities, and differences in implementation of PSS in ED settings.

## Methods

### Procedures

The NIDA CTN includes 16 academic research “nodes” across the nation which are linked with community treatment sites where pragmatic clinical research is performed. As part of the site selection process for the current multi-site clinical trial, all CTN nodes (n = 16) were sent an invitation for their affiliated clinical sites to complete and submit a SSS for the NIDA CTN study (CTN-0107, UG DA013727): Peer Intervention to Link Overdose Survivors to Treatment (PILOT), funded by the NIH HEAL Initiative℠. Invitations were sent via recruitment emails through the CTN listservs and announcements at weekly national Node Coordinator Meetings. A total of 11 sites completed the electronic survey, which included sites from 8 out of 16 nodes. All 11 SSSs were included in this analysis. SSSs were completed between November 2020 and January 2021 by site representatives and submitted directly to the lead research team at the Medical University of South Carolina, Metrics captured in the SSSs represent data from 2020. The IRB deemed the project as quality improvement and thus, informed consent was not required.

### Measures

The SSS included 40 questions obtaining information on PSS hiring, hours, funding, credentialing, pay, training certification, supervision, peer procedures and services provided. This information was collected in service of defining the treatment as usual condition and informing onboarding and budgeting for PSS in the parent study. Given the timing, additional questions surrounding COVID-19 operations were asked to assess for the impact of future outbreaks on study recruitment and operations. Questions about the strengths and barriers to providing PSS in the ED were assessed to better understand the feasibility of implementing the intervention within the ED workflow. The survey contained both quantitative inquiries and free text sections resulting in qualitative data [See Additional file 1].

### Data analyses

Free text questions on the SSS made up the qualitative data for this study. Quantitative data analysis consisted of descriptive and frequency statistics conducted in SPSS 28.0. Qualitative data analysis consisted of a qualitative content analysis [5], used to explore participants’ unique perspectives via the identification of themes/patterns that naturally emerge from the data and the systematic classification of these themes [11]. Specifically, a three-step inductive approach was utilized, in which each participant’s interview responses (i.e., raw data) were carefully examined to develop a comprehensive codebook to capture all possible themes emerging from the data. The codebook was then used by two independent coders to code and analyze each participant’s responses to the interview questions [5, 8]. Coders were able to apply more than one code to participant responses if applicable. Regarding reliability checks, 10% of the observations were double coded, and a Krippendorff’s Alpha Reliability Estimate [13] found reliability of 0.9261. Inter-rater discrepancies were discussed and resolved by the two independent coders. Finally, themes were refined, merged, and/or subdivided into sub-themes via collaborative discussion in multiple in-person meetings until a comprehensive codebook was developed. NVivo 12 software was used for qualitative data management and analysis.

## Results

Results from the SSSs have been compiled and are presented below based upon theme and descriptive information provided.

### Participants

Participants included 11 site-affiliated EDs in the US that completed SSS as part of the site selection process for the NIDA CTN PILOT study. Characteristics of sites are described in Table 1. Sites reported a wide range in number of patients presenting to the ED with a diagnosis of OUD or an opioid-related issue, ranging from 3 to 100 admissions per month (average 46 patients each in September and October 2020). Number of OUD- and opioid-related admissions were obtained via International Classification of Diseases (ICD) codes (64%), information in the EHR not specified (27%), or a combination of ICD codes and chart review (9%). Most sites (91%) reported having the ability to initiate medications for OUD (MOUD; methadone, buprenorphine, or naltrexone) prior to ED discharge, with a range of 0 to 25 patients initiated on MOUD in the ED each month (average 11.95 patients each in September and October 2020). Finally, sites reported a range of 62 to 500 opioid-related overdoses presenting to their ED in the past year (average 253), with a range of 2 to 40 (average 20) in September 2020 and 2 to 33 (average 20.8) in October 2020.

**Table 1:** Characteristics of included emergency departments

Site	State	Resident rotations	EM residency	Teaching designation	Location	Size	Annual ED volume	Adult trauma level
1	SC	No	No	Minor	Urban	Medium	36 k	II
2	WA	No	No	Non-Teaching	Urban	Medium	25 k	None
3	PA	Yes	No	Major	Urban	Large	52 k	I
4	UT	Yes	Yes	Major	Suburban	Large	75 k	I
5	OH	Yes	Yes	Major	Urban	Large	95 k	I
6	PA	Yes	No	Minor	Urban	Large	89 k	II
7	SC	Yes	Yes	Major	Suburban	Large	50 k	I
8	WA	Yes	Yes	Major	Urban	Large	75 k	I
9	CA	Yes	Yes	Major	Urban	Large	75 k	I
10	OH	Yes	Yes	Major	Suburban	Large	47 k	I
11	WV	No	No	Non-teaching	Rural	Small	15 k	None

The information in this table was completed by the sites including the authors on this paper, as well as by information available about each ED online. Teaching designation was defined by Liu et al. [19]. Size was determined by overall hospital bed number (< 100beds = small; 100–499 beds = med; 500 +  = large)

### Peer support specialist hiring, hours, funding, credentialing, pay

PSS had been implemented at the 11 ED sites for a range of 5 months to 4 years (mean = 24.17 months). Sites reported that they had a mean average of 2.54 full-time (range = 0–5; median = 3) and 1.09 part-time (range = 0–6; median = 0) PSSs, with most sites (82%) having at least one full-time PSS. More than half (64%) of the sites reported their PSSs were affiliated with local recovery organizations, such as Alcoholics Anonymous (AA), Narcotics Anonymous (NA), Faces and Voices of Recovery (FAVOR) or other local recovery organizations.

Sites anticipated current funding for PSSs to continue for 1 to 3 years (mean = 16 months). PSSs’ employment was financially supported through a combination of grant funds (55%), hospital funds (46%), peer recovery organizations (27%), or other sources (18%). The majority (82%) of PSSs were paid hourly with a range of $13 to $23 per hour (mean $15.10 per hour); the two sites with salaried PSSs paid salaries of $34,000 to $36,000 per year. Most of the PSSs (82%) were offered benefits in addition to wages, and one site offered additional stipends and mileage reimbursement for the PSSs.

The largest discrepancy among sites was in relation to the hours and availability of PSSs in the hospital. Three of the sites (27%) reported that PSSs were available 24-h/day, 7 days/week. All other sites varied tremendously, with two sites (18%) having PSSs available on the weekends (approximately 12 h/day). Five sites (46%) had PSSs available Monday through Friday, 8:00 am to 5:00 or 6:00 pm, with one site having PSSs working until 8:00 pm during the week. A slight majority of PSSs worked shifts only (46%), whereas 36% were on-call only, and 18% worked both shifts and on-call.

### Peer training, certification, and supervision

There were differences across sites in PSS qualifications, background, and training as well as in supervision and ongoing training. All sites required a high school diploma for PSSs, although only four sites (36%) required PSS certification. The description of type of ongoing clinical supervision and training of PSSs differed widely among sites, with supervision of PSSs being conducted by a range of professionals including ED supervisors, nurse care managers, senior PSSs, behavioral nurse practitioners, grant program managers, attending physicians, or an ED peer director.

### Peer procedures and services provided (including COVID-19 operations)

Five of the sites (46%) reported having a hospital-based peer notification system in place. With 3 sited describing a hospital-based overdose notification system, primarily consisting of a phone call or page to the PSS from a social worker or ED staff member. One site notified PSSs via flag in the EHR. Most sites (73%) reported having EHR access, most commonly the EPIC^®^ system.

The type of peer services for SUD provided in the EDs varied significantly by site and were self-defined by each of the sites. The sites described their services as general “recovery support” (9 sites), SBIRT or screening/referral services (6 sites), peer-involved buprenorphine fast-track programming (3 sites), “warm-handoff” or assistance with treatment entry (3 sites), peer coaching or consultation (2 sites), education (2 sites), mentorship (1 site), resource navigation (1 site), and advocacy (1 site). The majority of sites had peer involvement in naloxone distribution (64%), with those sites relaying their PSSs were providing naloxone to all patients that have overdosed and/or OUD (see Table 2). Only 27% of sites had peer involvement with EDIB despite 91% of sites indicating that EDIB was available.

**Table 2:** Services provided by peer support specialists

Peer services provided	Percentage of sites providing this service %
Recovery support	82
SBIRT or screening/referral services	55
Peer-involved buprenorphine "fast-track" programming	27
Warm handoff or assistance with treatment entry	27
Peer coaching	18
Education	18
Mentorship	9
Resource navigation	9
Advocacy	9
Naloxone distribution	64
Services post-ED discharge	91

Most sites (91%) reported that PSSs provided some services to patients with SUD following ED discharge, ranging in duration from one day to 12 months after discharge. Six of the sites (55%) initiated PSS follow up between one day and 1 week following ED discharge, while 27% conducted a 3-month and 6-month follow-up, and 9% provided a 12-month follow-up. Most sites reported either providing the patient with a referral (45%) or “bridge” prescription (45%) with a next-business-day appointment for ongoing care, and one site had PSSs attend the next appointment with the patient.

During the COVID-19 pandemic, most sites remained open for in-person peer support services with 36% providing only in-person services and 64% providing a combination of in-person and virtual services. Only one site closed their peer services completely where PSSs did not see patients for a period of time during the pandemic.

### Strengths and barriers to providing peer services in the ED

Sites identified a variety of facilitator and barriers pertaining to the implementation and maintenance of the peer support programs within their settings. The primary facilitators for having and maintaining PSSs in the ED included having strong ongoing relationships with treatment facilities and community organizations, similar EDIB procedures across clinics and referral settings, existing MOUD programs, ongoing research involvement and experience, multi-system teams, and joint motivation to treat and assist in the community. The primary barriers cited in implementing and maintaining successful PSS programs included lack of outpatient referral sources, barriers to initiating MOUD in the ED, stigma at the clinician and system level, and lack of ongoing availability of PSSs due to short-term grant funding.

## Discussion

This descriptive report from 11 ED settings across the US reveals that PSSs have been integrated into diverse ED settings and are serving ED patients with SUDs and their providers. There were important themes and similarities found among the 11 sites participating in the SSS: (1) programs have time-limited funding; (2) PSSs provide on-going services after ED discharge, (3) PSSs are involved with naloxone distribution but less with MOUD initiation, (4) lack of “automated” peer notification system, and (5) PSS services continued through the COVID-19 pandemic.

These results show that in these 11 ED sites, incorporation of PSSs into ED settings has largely been supported through grant-funding (55%), though importantly, nearly half of the sites also reported receiving some hospital funding. Furthermore, the mean time of anticipated funding was only 16 months. Time-limited funding was identified as a significant barrier to implementation and maintenance of ED PSS services, and this finding adds to the existing literature of barriers for these programs [16]. As SOR and STR grants eventually conclude, there will be an ongoing need for medical facilities and the peer support field to evaluate sustainability plans for retaining PSSs in medical settings such as EDs.

Secondly, PSSs at most ED sites continued contact with patients after ED discharge, suggesting that communication with patients outside of the ED visit is valued and may facilitate outpatient follow-up and ongoing treatment. Prior studies have also described continued contact with patients after ED discharge through post-overdose outreach programs [2]. When considering PSS workflow and workload, this time spent attempting and executing ED follow-up contact and this regular work outside of the ED setting should be accounted for. Given that duration and intensity of contact after discharge varied appreciably among sites, further evaluation of outcomes impacted by PSS contact post-ED discharge as well as ideal frequency and duration of contact is warranted.

The majority of programs had PSS involvement with naloxone distribution, but only a minority of PSSs were involved with EDIB programs. While this survey did not specifically ask what the PSS’s involvement in EDIB programs entailed, previous work has indicated that the PSS can be helpful in identifying potential candidates and assisting with linkage to continued care after the ED visit [4, 21]). Peer recovery services have also been shown to be beneficial in connecting patients to MOUD through bridge clinics [30]. It is unclear whether buprenorphine was “available” via in-ED dosing and/or buprenorphine prescriptions at discharge, pointing to an important area needing further clarity. This also highlights an important potential opportunity for growth and facilitation for both ED overdose education and naloxone distribution (OEND) and EDIB. EDIB has been shown to double treatment retention at 30 days [9], but one of the identified barriers to implementing EDIB in this and other studies is lack of a process for linkage to outpatient treatment following EDIB [15]. In this SSS, sites noted as a facilitator the well-established ongoing relationships between PSSs and treatment facilities and community organizations. The ability of the PSSs to connect and bridge transitions of care in the ED highlights a prime opportunity to enable PSSs to facilitate EDIB, OEND, and follow up care coordination.

Interestingly, a minority of sites had a peer notification system in place, and those that did have a system primarily relied on ED staff to call or page the PSS. Prior work has demonstrated that automated referrals (based on prior interactions with PSS, SUD screening tools, buprenorphine and/or naloxone prescription and/or administration, or ICD codes) to peer support services have been quite successful [18, 20]). Broader use of these automated notification systems may be an opportunity for improvement for many ED-based PSS programs.

In all but one site, PSSs remained available to patients in the ED, either in-person, virtually, or both, during the COVID-19 pandemic, suggesting their important integration as front-line health care workers, especially during the peak of the pandemic. Further, PSSs have been logistically integrated into the medical care team, as evidenced by PSSs having access to the hospital EHR. These steps of integration of the PSS workflow into the medical team and EHR is an important step for efforts to advance PSS billing and reimbursement for services, as documentation in the EHR is the most common means for billing within the healthcare industry.

Other notable differences between programs included variability in peer supervision, peer certification requirements, and source of funding. Given the recent and rapid growth of the PSS role in healthcare settings [22] and the grassroots development of peer services during a time of increased federal funding to address the opioid crisis in front-line healthcare settings, such variability is not surprising. Future studies should examine peer supervision, peer certification, and funding sources to determine the most effective and cost-effective way to create and sustain PSS programs within ED settings while also ensuring reliable consistency of service delivery, especially if funding for opioid-related initiatives decreases.

Peer certification programs exist, but they vary state-to-state in content, requirements, and duration. Only recently have national standards been created [29]. Likewise, training in peer supervision is also being developed across various settings, but remains in preliminary stages. Our results highlight that PSS supervision for ED-based peers is being delivered by providers of varying disciplines, suggesting that efforts of organizations to fit a relatively new hospital-based professional role into an existing ED/hospital structure has resulted in heterogeneous implementation. Additionally, the significant variation in the job requirements and training to be a PSS, including the requirement for peer certification and what organization provided that certification, highlights the possibility that the growth and demand for PSSs in ED settings outpaced the availability of peer certification and formalized supervision structure in some states. These findings of heterogeneity in training and peer certification requirements in the ED setting are consistent with findings from peers being used in non-ED settings [6]. Whether a more uniform certification process for PSSs is needed and whether uniform certification would add or subtract value to the field is an area of debate. Notably, one of the strengths of the PSSs is that they can be more accessible, flexible, and often respond more quickly and innovatively to evolving local demands of the opioid crisis than large, formalized, heavily regulated healthcare systems or governing bodies. Future research should examine these certification requirements by state.

Given the descriptive and retrospective nature of this study, there are several study limitations: (1) Generalizability: There are a small number of sites that self-selected to participate based on perceived eligibility for the clinical trial; therefore we did not receive information from sites that chose not to participate nor sites that were not affiliated with a CTN node. Although one of the primary aims of the CTN is to perform clinical research in real-world settings, it is possible that sites affiliated with a CTN node may differ from typical sites. Furthermore, we do not have any information on how sites that did not participate or did not respond, and therefore cannot describe how those sites may be different than sites that did respond. Each of these factors affects generalizability. (2) Indirect data with limited detail: The SSSs were developed to gather information about eligibility and matching for site selection for a clinical trial. Therefore, self-reported answers were provided and not objectively verified. Therefore, the accuracy of the information provided cannot be confirmed, and specific details regarding topics such as the nature of peer support supervision or how specifically PSSs are involved with EDIB were not provided. However, the SSS data has resulted in 3 successfully operationalized sites for the PILOT study, where detailed data about peer training, peer supervision, and the nature of the interaction between participants and PSSs is being gathered.

## Conclusions

This descriptive analysis of 11 ED sites providing PSS services highlights that PSSs, supported through federal grant funding and hospital systems, have been incorporated into EDs across the US and have provided integral services to people with SUD during the height of the opioid crisis and COVID-19 pandemic. There was important consistency among sites in the involvement of PSSs in facilitation of transitions of SUD care, coordination of follow-up after ED discharge, and naloxone distribution. There were also similarities across programs that highlight areas for growth including increased peer involvement in ED-initiated MOUD, implementation of automated peer notification systems, and addressing time-limited funding of many ED PSS programs. There was variability in peer credentialing requirements, training and supervision. The grass-roots and state-defined implementation [24] of PSSs in medical settings has allowed for PSSs to respond creatively to the unique needs of their communities with the given resources of that community, but has led to understandable state-to-state variability in PSS training, certification, role, supervision, services delivered, and sources of funding for PSS services. This variability creates challenges in evaluation of overall efficacy of peer services, development of national guidelines, and uniform approval of service reimbursement necessary to sustain PSSs in medical settings without grant funding. Although variability in the scope of practice of PSSs in different settings can be expected, a basic core identity for PSSs that includes who trains, supervises, and ensures competency of PSSs still needs further clarity.

### Supplementary Information

**Additional file 1. **Site Selection Survey.

## Data Availability

The datasets during and/or analysed during the current study available from the corresponding author on reasonable request.
