# Supplementary material for: Characterization of peer support services for substance use disorders in 11 US emergency departments in 2020: findings from a NIDA clinical trials network site selection process
Source: Addict Sci Clin Pract. 2024 Apr 8;19:26. doi: 10.1186/s13722-024-00453-x (PMC11003047; doi:10.1186/s13722-024-00453-x)
Supplement: Supplementary file 1 — Additional file 1. Site Selection Survey. [file 13722_2024_453_MOESM1_ESM.docx]

**CTN-0107 Peer Intervention to Link Overdose survivors to Treatment (PILOT)**

**Site Selection Questionnaire**

On behalf of the Southern Consortium Node of the [Clinical Trials Network](https://www.drugabuse.gov/about-nida/organization/cctn/clinical-trials-network-ctn) of the National Institute on Drug Abuse, we thank you for considering this site selection questionnaire. [CTN-0107](https://www.drugabuse.gov/about-nida/organization/cctn/ctn/research-studies/peer-recovery-support-bridge-to-treatment-overdose-survivors-nih-heal-initiative) will evaluate a peer-delivered intervention for overdose survivors presenting to the emergency department (ED). Though completion of this form does not signify your commitment or agreement to participate in the study as a site, it may help us understand your potential interest and eligibility. We recognize that some of these questions may require obtaining additional information from your ED/hospital or clinic providers. If you are unable to complete any of the questions below, please indicate that you cannot answer this question with a brief explanation. Please reach out to the study coordinator, National Project Director, or the Principal Investigator with any questions or concerns. Thank you!

**Important Dates**

- **Please inform us of your *intent* to complete the survey by December 15, 2020.**
- **Please *submit* the completed site selection survey by January 12, 2021.**
- **We plan to schedule site interviews in the weeks of 1/18/21-1/29/21.**

**Study Overview**: The primary objective of the [CTN-0107](https://www.drugabuse.gov/about-nida/organization/cctn/ctn/research-studies/peer-recovery-support-bridge-to-treatment-overdose-survivors-nih-heal-initiative) (PILOT) study is to improve outcomes for individuals who present to an ED after surviving a non-fatal OD involving opioids (NFOO). We will evaluate the preliminary effectiveness of a specialized peer recovery intervention tailored for NFOO survivors, called **P**eer **I**ntervention to **L**ink **O**verdose survivors to **T**reatment (PILOT) compared to treatment as usual (TAU) in the ED to determine if the PILOT intervention reduces overdose risk behaviors.

The PILOT study is a prospective, randomized, controlled preliminary trial that will recruit approximately 150 NFOO survivors from three ED sites in the US. Participants will be approached and randomized during their ED visit. To be eligible, participants must have had an NFOO in the past 30 days. Participants will be randomized to the PILOT intervention or TAU. Participants will complete study visits at Months 1, 3, and 6, as well as a follow-up visit at Month 7. Recruitment will last 12 months. The primary hypothesis is that those randomized to PILOT will have reduced overdose risk behaviors compared to those randomized to TAU based on an overdose risk behavior scale. This trial will allow for the rigorous testing of the PILOT intervention to determine if this program is effective in reducing harm among those with a recent NFOO

| **Organization Name:**  Address:  CTN Node:  Node Contact: |
| --- |
| **Site-affiliated ED Name:**  Address:  ED Clinical Champion (if known), & current position: |
| **Site PI:**  Phone:  Email: |
| **Person completing the survey** (name/title/affiliation): |

**PEER SUPPORT SPECIALIST INFORMATION**

1. One of the site requirements for CTN 107 is to have peer support specialists currently working within an affiliated Emergency Department (ED).

Please describe what peer services are currently provided in your affiliated ED: (e.g. SBIRT, Buprenorphine Fast-Track protocols, Recovery Support services, other):

2. Please describe the current referral process for ED patients with SUD or overdose to community treatment agencies (substance abuse, mental health, etc):

3. Do the ED peers currently provide **any** services or follow-up to ED patients **after** they leave the ED?

Yes No

3a. If **yes,** please detail type, duration and frequency of post-ED contact:

4. Which agency/institution employs the ED peer specialists (check all that apply and provide detail below)?

Hospital Peer Recovery Organization Grant Other

5. How long is the current funding source guaranteed for the ED peers?_______________________________

6. How long have peer recovery specialists been providing services in this ED? ______________

7. Are the ED peer recovery specialists affiliated with any local recovery organizations (e.g. FAVOR)?

Yes No

If **yes,** which organization and about how large is that locally affiliated organization (total number of peers)?

8. How many peers (FTE) are currently hired to working in the ED? _____

What hours do peers cover in the ED? __________

9. How many ED peers are full-time?______ Part time?_________

10. Please describe the salary structure for your ED peer support specialists.

How many peers are paid hourly?______ Hourly rate?_____________

How many peers are salaried? __________ Salary range and fringe? ___________

Do any peers receive benefits? Yes No

Estimated total cost per employee? ________________

11. Do ED peers have access to the Electronic Medical Record (EMR)? Yes No

If **yes,** what EMR system does your ED site use (e.g. Epic, Cerner, Centricity, etc)? ______________ Do the peer recovery specialists use the same or separate EMR than the ED? Same Separate

12. Do ED peers have shifts where they are present in the ED or are they called into the ED on as as-needed basis (or both)? Shifts On-call Both

13. Please describe who provides supervision to the peers and how/frequency?

14. This study will fund the hiring of up to 4 FTE peer recovery specialists that would exist in addition to your currently employed ED peers (funded outside of the study). The 4 FTE research intervention peers would work solely as research intervention peers engaging with OD survivors, so they would be separate employees from the current ED peers. Please describe the hiring and hospital credentialing process (if required) for peer recovery specialists in your ED site. Please specify how much time would be required for advertising, interviewing, and on-boarding of up to 4 additional FTE peer specialists.

15. Are the ED peer recovery specialists required to have any certain qualifications such as a bachelor’s degree or certification as a peer support specialist? Please describe.

15. Please describe the space in/near the ED that would be available for research staff on this study (4 study peer specialists and 1 FTE RA). Please note that the study peers would not necessarily need to be in the ED at all times and could be called as potential participants are identified.

16. Please describe how the ED peer specialists at your site have been delivering care/referrals during COVID-19 pandemic. Are they currently working remotely, etc.? If not working remotely now, have the peers worked remotely since pandemic onset? Please explain.

17. Was there a period of time since pandemic-onset where peers were ***not*** allowed to access the ED, even remotely?

Yes No If **yes,** please describe:

**SITE INFORMATION**

Defining a site: This study requires that participants are recruited from an ED. There may be more than one way to define a study site (i.e., academic institution, hospital, community treatment program, etc.).

18. Please describe the current relationship between your institution, node, ED/hospital system and peer recovery organization and what organization might best represent the “site.”

19. Which organization would perform the hiring and supervising?

20. Given the description of the study, how do you envision this working within your site? How would it work for peers to identify study participants and communicate with the research staff given your existing processes? How do you foresee meeting recruitment goals of 4-5 subjects/month (from those presenting with overdose and those with OUD/opioid related issue with overdose in past 30 days)?

21. As described on the first page, participating sites will randomize participants into two different conditions (TAU or PILOT).

21a. Is your site willing to randomize participants into the 2 study conditions? Yes No

21b. Do you foresee current ED peers being willing to work collaboratively with the new research peers? Yes No

22. Does your ED-affiliated site have a peer notification system for when patients arrive who have SUD?

Yes No If **yes,** please describe briefly. If **no**, how do your peers identify patients with SUD?

24. Does your ED-affiliated site currently screen for or have a notification system specifically for overdoses?

Yes No If **yes,** please describe briefly

25. Does your site-affiliated ED provide Narcan to patients presenting to the ED? Yes No

If **yes,** to which populations (all SUD, all OUD, post-OD only)?

If **no,** would you will be willing/able to provide a Narcan kit if not part of your TAU? Yes No

26. Other than Narcan, are there any other post-overdose specific treatments or follow-ups being offered to overdose survivors in your site-affiliated ED? Yes No

If **yes,** please detail:

27. Should the study need to recruit from outside the ED, please list any community partner sites from which you may potentially recruit study participants with a history of recent drug-related overdose (within the past 30 days).

**EMERGENCY DEPARTMENT DATA:**

29. Is your site capable of obtaining data on ED admissions for opioid-related admissions and overdoses?

Yes No

30. How many patients presented to your site-affiliated ED with a diagnosis of OUD or opioid-related issue in:

September 1-30, 2020:

October 1-31, 2020:

A. Please describe how this data was obtained (check all that apply):

EMR search system such as slicer/dicer (please provide search terms below)

ICD-10 (please provide codes below) chart review other (please describe below)

31. Does your ED have capability to initiate patients on medications for OUD (MOUD; methadone, buprenorphine or naltrexone) prior to discharge? Yes No

A. If **yes,** how many patients were initiated on MOUD in the ED in:

September 1-30, 2020:

October 1-31, 2020:

Please describe how this data was obtained:

B. If **no**, is your ED capable of initiating patients onto MOUD during their visit? What are the barriers to initiating MOUD?

32. Please provided the number of ED admissions for opioid-related overdoses at your ED site:

In the last year (October 2019-October 2020):_______________

September 1-30, 2020:______________

October 1-31, 2020 _________________

A. Please describe how this data was obtained (check all that apply):

EMR search system such as slicer/dicer (please provide search terms below)

ICD-10 (please provide codes below) chart review other (please describe below)

B. From this data, is your site able to describe the days of the week and time of arrival and/or time of discharge of suspected overdose patients? Yes No

If YES, please describe the peak days of week and times for overdose victims to arrive in your ED:

**RESEARCH EXPERIENCE/IRB**

33. Is your site-affiliated ED currently participating in any other clinical trials that recruit individuals with a recent history of drug related overdose? Yes No

A. If **yes**, please briefly describe.

34. This study will provide funding for up to 1 FTE of Research Assistant (RA) time. Does your site currently have RAs already actively working in the ED for other studies, and if so what times/days?

35. Please list any research your site has conducted in the past 5 years. If more than 3, please list those most similar to this study (eg utilizing peers or in the ED) and/or any CTN studies.

| Study Name | Sponsor | Study Population | Target Randomization | Actual Randomization |
| --- | --- | --- | --- | --- |
|  |  |  |  |  |
|  |  |  |  |  |
|  |  |  |  |  |

36. Does your site have an IRB? Yes No

36a. The IRB at the Medical University of South Carolina, has full AAHRPP accreditation, and will serve as the single IRB for this study. Would your site be able to rely on MUSC? If so, would your institution have additional reviews that would need to occur? Please describe.

36b. If **no,** has your site affiliated with another IRB in previous research studies? Yes No

36c. If **yes or no,** is your site a member of SmartIRB or willing to become a member? Yes No

**BARRIERS TO STUDY IMPLEMENTATION:**

37. Are you aware of any upcoming events at your ED site such as relocation, adopting a new EMR, space modifications (especially related to COVID), legal/regulatory changes, or funding changes, which may occur during the course of the study? Yes No

If **yes,** please explain:

38. Are there any current hospital-based, community, or state-level interventions that exist in the catchment area of the site-affiliated ED that target OD survivors (E.g. EMS conducting post-OD home visits, etc)?

Yes No If **yes,** please explain:

39. Are you aware of any new or planned community or state-level interventions or initiatives being planned to target overdose survivors which may occur during the course of the study? Yes No If **yes,** please explain:

40. If needed/requested would your site be willing/able to provide prospective data (e.g. 1 week of screening for overdoses in ED)? Yes No

**Thank you very much for completing this survey!**
